# Supplementary material for: The eukaryotic translation initiation factor eIF4E harnesses hyaluronan production to drive its malignant activity
Source: eLife. 2017 Nov 7;6:e29830. doi: 10.7554/eLife.29830 (PMC5705209; doi:10.7554/eLife.29830)
Supplement: Transparent reporting form [file elife-29830-transrepform.docx]

***eLife’s* transparent reporting form**

We encourage authors to provide detailed information *within their submission* to facilitate the interpretation and replication of experiments. Authors can upload supporting documentation to indicate the use of appropriate reporting guidelines for health-related research (see [EQUATOR Network](http://www.equator-network.org/%20)), life science research (see the [BioSharing Information Resource](https://biosharing.org/)), or the [ARRIVE guidelines](http://www.plosbiology.org/article/info:doi/10.1371/journal.pbio.1000412) for reporting work involving animal research. Where applicable, authors should refer to any relevant reporting standards documents in this form.

If you have any questions, please consult our Journal Policies and/or contact us: [editorial@elifesciences.org](mailto:editorial@elifesciences.org).

**Sample-size estimation**

- You should state whether an appropriate sample size was computed when the study was being designed
- You should state the statistical method of sample size computation and any required assumptions
- If no explicit power analysis was used, you should describe how you decided what sample (replicate) size (number) to use

Please outline where this information can be found within the submission (e.g., sections or figure legends), or explain why this information doesn’t apply to your submission:

-Sample size is mentioned in Materials and Methods section.

-Statistical method, standard deviation, means and p-values are mentioned in Figure legends and/or Material and Methods section.

-For analysis of animal specimens, we used the group of animals treated and untreated from Cancer Research paper, where a two-way ANOVA test determined a difference in number and size of METs between these groups. We analyzed tissue blocks from these animals and thus, this is a retrospective study.

-For cell line experiments, we used the standard of at least three biological replicates done in technical triplicate.

-For AML samples, our study was exploratory to determine whether there would be any difference in HA levels between the groups. We observed substantial differences in the specimens versus normal as assessed by Student t-test. P-values and standard deviations.

-No explicit power analysis was required.

**Replicates**

- You should report how often each experiment was performed
- You should include a definition of biological versus technical replication
- The data obtained should be provided and sufficient information should be provided to indicate the number of independent biological and/or technical replicates
- If you encountered any outliers, you should describe how these were handled
- Criteria for exclusion/inclusion of data should be clearly stated
- High-throughput sequence data should be uploaded before submission, with a private link for reviewers provided (these are available from both GEO and ArrayExpress)

Please outline where this information can be found within the submission (e.g., sections or figure legends), or explain why this information doesn’t apply to your submission:

-All experiments were performed at least three independent times as stated in Figure legends.

-Technical replicates when the same sample is analyzed in parallel while Biological replicates when the experiment is repeated on different occasions using different cells for instance.

-All experiments with p-values were biological replicates. In experiments with primary patient samples the replicates were technical.

-On the rare occasion outliers were found in RT-qPCR cycle numbers to be clearly variant, a Dixon’s Q-test was performed and if appropriate the number was removed. There were no outliers for any of the other experiments.

-No high-throughput analyses were performed.

**Statistical reporting**

- Statistical analysis methods should be described and justified
- Raw data should be presented in figures whenever informative to do so (typically when N per group is less than 10)
- For each experiment, you should identify the statistical tests used, exact values of N, definitions of center, methods of multiple test correction, and dispersion and precision measures (e.g., mean, median, SD, SEM, confidence intervals; and, for the major substantive results, a measure of effect size (e.g., Pearson's r, Cohen's d)
- Report exact p-values wherever possible alongside the summary statistics and 95% confidence intervals. These should be reported for all key questions and not only when the p-value is less than 0.05.

Please outline where this information can be found within the submission (e.g., sections or figure legends), or explain why this information doesn’t apply to your submission:

-Statistical method and p-values are mentioned in Figure legends and Materials and Methods section.

-Average or Mean values and SD are also indicated whenever used under Figure legends and Materials and Methods section.

-Exact p-values are included in Figures where appropriate.

(For large datasets, or papers with a very large number of statistical tests, you may upload a single table file with tests, Ns, etc., with reference to sections in the manuscript.)

**Group allocation**

- Indicate how samples were allocated into experimental groups (in the case of clinical studies, please specify allocation to treatment method); if randomization was used, please also state if restricted randomization was applied
- Indicate if masking was used during group allocation, data collection and/or data analysis

Please outline where this information can be found within the submission (e.g., sections or figure legends), or explain why this information doesn’t apply to your submission:

-Not Applicable.

**Additional data files (“source data”)**

- We encourage you to upload relevant additional data files, such as numerical data that are represented as a graph in a figure, or as a summary table
- Where provided, these should be in the most useful format, and they can be uploaded as “Source data” files linked to a main figure or table
- Include model definition files including the full list of parameters used
- Include code used for data analysis (e.g., R, MatLab)
- Avoid stating that data files are “available upon request”

Please indicate the figures or tables for which source data files have been provided:

-Not Applicable.
